# Arterial Thrombosis Diagnosed With Point-of-Care Ultrasound

**DOI:** 10.7759/cureus.69357

**Published:** 2024-09-13

**Authors:** Andrew A Gonzalez, Daniel S Brenner

**Affiliations:** 1 Vascular Surgery, Indiana University School of Medicine, Indianapolis, USA; 2 Emergency Medicine, Indiana University School of Medicine, Indianapolis, USA

**Keywords:** atrial thrombosis, covid 19, general and vascular surgery, limb ischemia, pocus (point of care ultrasound)

## Abstract

Acute arterial thrombosis is a rare but dangerous condition that requires rapid diagnosis and treatment to reduce the risk of amputation. SARS-CoV-2 is associated with an increased risk of arterial thrombosis. Point-of-care ultrasound (POCUS) can facilitate rapid bedside evaluation for both venous and arterial thrombosis and expedite treatment in these time-sensitive diagnoses. Although POCUS diagnosis of venous thrombosis is well studied, few cases of POCUS diagnosis of arterial thrombosis have been reported. We present a case in which acute SARS-CoV-2-associated arterial and venous thromboses were diagnosed at the bedside utilizing POCUS, which led to expedited operative management and limb preservation.

## Introduction

Acute limb ischemia is a time-sensitive and highly morbid condition caused by occlusive thrombosis or embolism in an artery, with resultant downstream tissue ischemia. It is relatively uncommon (4.16/100,000 person-years) [1] and represents the extreme end of the broad spectrum of ischemic limb disease. As rapid intervention is essential in preventing tissue loss, expedited evaluation for acute arterial and/or venous thrombosis is essential for effective patient care in patients with a high pretest probability for these conditions. While the gold standard imaging study for arterial thrombosis is arteriography, logistical delays in performing arteriographic studies can delay diagnosis and treatment, and previous data have demonstrated the utility of duplex Doppler as a viable first radiological study for limb ischemia. [2] A brief point-of-care ultrasound (POCUS) examination evaluating the proximal deep vasculature in the lower extremity can expedite diagnosis and treatment in patients who have signs and symptoms of acute limb ischemia. Prior work has demonstrated that POCUS is effective in screening for proximal venous thrombosis in the emergency department and expedites diagnosis [3]. While arterial studies are less commonly performed by emergency physicians, case reports of POCUS aiding in the diagnosis of arterial thromboses have also been published [4-6].

We present a case of SARS-CoV-2-associated acute arterial and venous thrombosis that was identified with POCUS during the initial evaluation of the patient, leading to expedited operative management.

## Case presentation

A 59-year-old man with HIV (CD4 count 669 on elvitegravir-cobicistat-emtricitabine-tenofovir) and diabetes mellitus (on metformin and sitagliptin) presented to the Emergency Department with leg pain. He had been diagnosed with SARS-CoV-2 two weeks prior and had been quarantined at home with mild respiratory symptoms. He did not receive steroids, monoclonal antibody therapy, or any other SARS-CoV-2-specific medications and continued his home medications without any missed doses. His respiratory symptoms improved, but he developed sudden onset, severe pain in his right leg, which prompted his presentation to the emergency department. His pain was similar in nature to prior episodes of sciatica but significantly worse. He denied any trauma, injection drug use, or history of thromboembolism.

**Figure 1:**
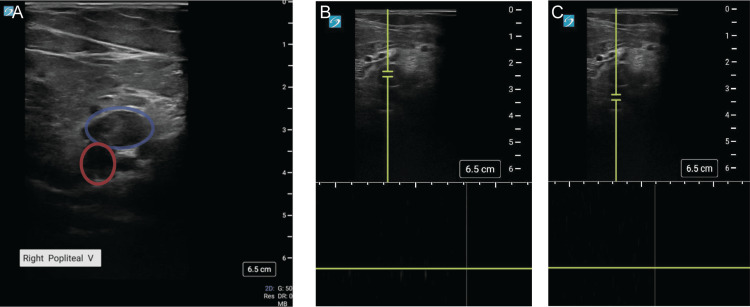
Arterial and venous thrombosis A) Still image of popliteal fossa demonstrating popliteal vein (blue circle) and popliteal artery (red circle). Both vessels contain mixed-echogenicity clots in the lumen. B) Spectral doppler showing no flow in the popliteal vein. C) Spectral doppler showing no flow in the popliteal artery.

His initial examination was notable for sinus tachycardia with a rate of 130 beats per minute. His right leg was colder than the left, and he did not have palpable dorsalis pedis or tibialis posterior pulses. He had reduced sensation in the right plantar and dorsal foot, as well as decreased strength in dorsiflexion and plantar flexion. With history and physical examination findings concerning limb ischemia, a POCUS examination was performed at the bedside using a Sonosite PX machine (Fujifilm Sonosite, Bothell WA) with an L12-3 linear transducer. The common femoral veins were compressible, and the arteries were pulsatile with a normal color Doppler flow signal. The popliteal vein was non-compressible, with mixed-echogenicity clot visible in the lumen and no evidence of flow on color or spectral Doppler (Figure 1A, 1B blue circle, Video 1). The popliteal artery was also noted to be non-pulsatile and lacked a signal with both color flow and spectral Doppler flow, consistent with an arterial thrombus (Figure 1A, 1C red circle, Video 2).

**Video 1:** Compression demonstrating non-compressible popliteal vein and non-pulsatile popliteal artery suggestive of arterial and venous thrombosis

**Video 2:** Color doppler demonstrating lack of flow in the popliteal artery and vein suggestive of arterial and venous thrombosis

**Figure 2:**
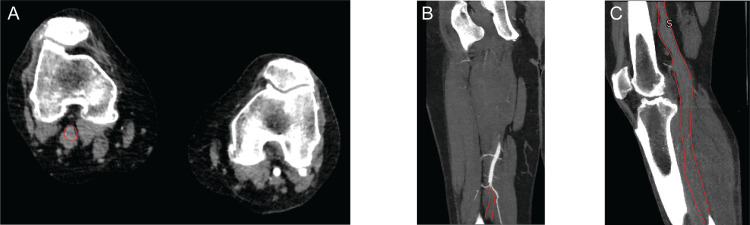
CTA confirming arterial thrombosis A) Axial CTA demonstrating lack of contrast opacification in the right popliteal artery (outlined in red). B) Coronal CTA demonstrating an abrupt cutoff of contrast opacification in the distal superficial femoral artery (thrombosed portion outlined in red). C) Sagittal CTA demonstrating lack of contrast opacification in the popliteal artery and the tibial artery (outlined in red)

Given evidence of acute limb ischemia with an arterial thrombus, heparin therapy was initiated, and the vascular surgery team began preparations for emergent operative intervention. As the operating room was being prepared, a CTA confirmed the diagnosis of popliteal arterial thrombosis (Figure 2, thrombosed vessels outlined in red) and revealed additional more proximal occlusions of the common and external iliac arteries as well. The patient underwent emergent thrombectomy and fasciotomies, but emergency amputation was not necessary. The patient was subsequently discharged on an oral anticoagulant with residual sensory loss to his lateral calf and mild motor deficits that did not prevent ambulation. Two years after the ischemic event, the patient had regained complete function and developed no further thrombotic events. His extended hypercoagulability workup was unremarkable.

## Discussion

**Figure 3:**
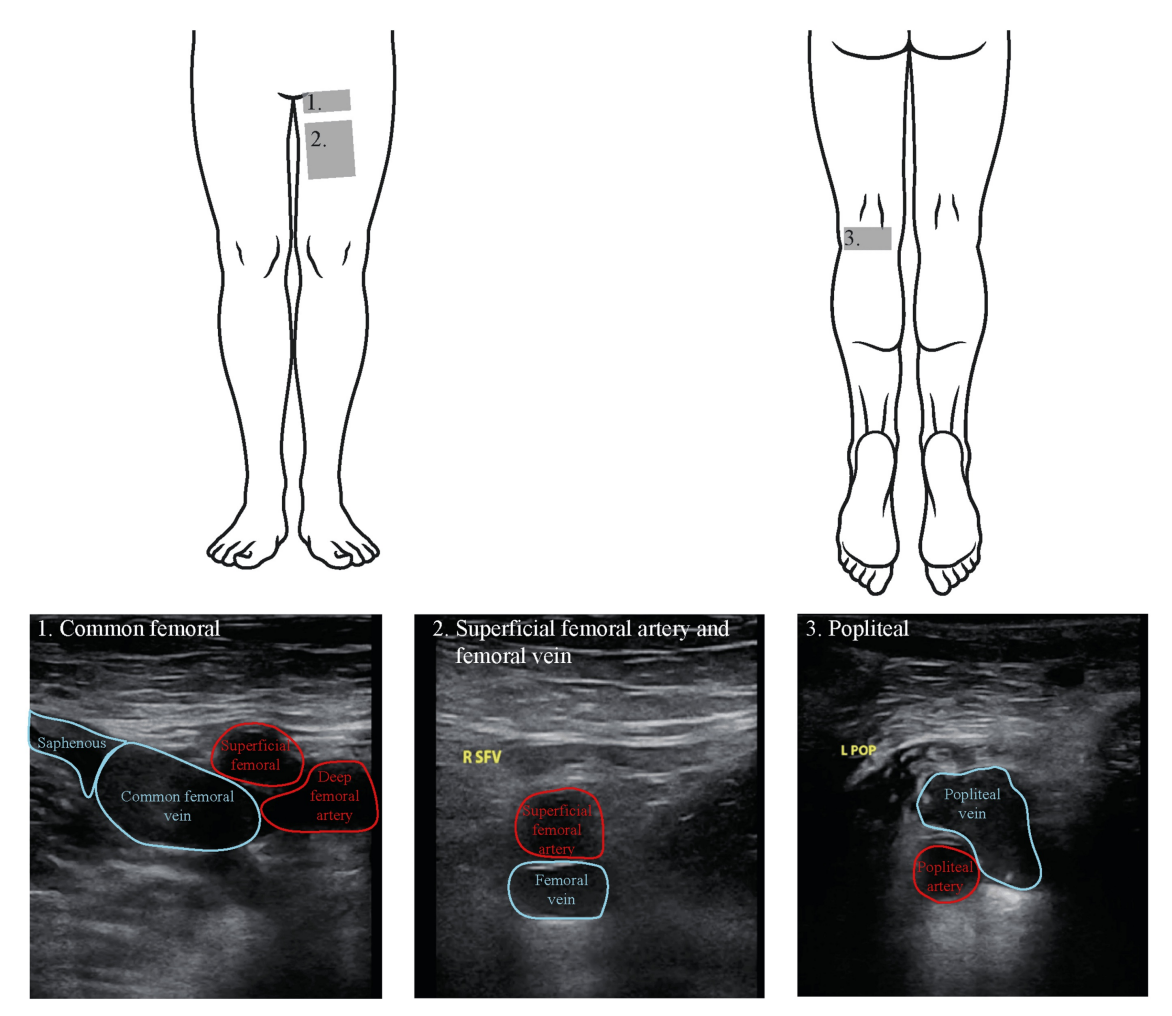
Schematic of leg vasculature for evaluation of proximal deep vein thrombosis Box 1 marks the general location of the common femoral vasculature, Box 2 marks the general location of the superficial femoral vasculature, and Box 3 marks the general location of the popliteal vasculature.

POCUS is a rapid and effective diagnostic tool for detecting both peripheral venous [3] and arterial pathology [5,6]. Unlike CTA or radiology-performed ultrasound, POCUS is a bedside test that can be performed without delay in the setting of critical illness. Case series have demonstrated the use of POCUS in the evaluation of vascular pathology, including arterial thrombus, expanding hematoma, infection, arteriovenous malformations, and trauma [7,8]. The most commonly used Emergency Department protocol assessing for deep venous thrombosis evaluates the common femoral, femoral, and popliteal veins because thrombosis in these vessels is known to have the highest risk of embolizing to the lungs [9]. This approach demonstrated in Figure 3 visualizes the common femoral vasculature at its junction with the great saphenous vein (box 1), the femoral vein and superficial femoral artery in the mid-thigh (box 2), and the popliteal vasculature in the popliteal fossa (box 3). While this is an efficient protocol that is validated for venous thrombosis, POCUS vascular evaluation can be adapted to any region of concern as guided by the area of pain or swelling in individual patients.

**Figure 4:**
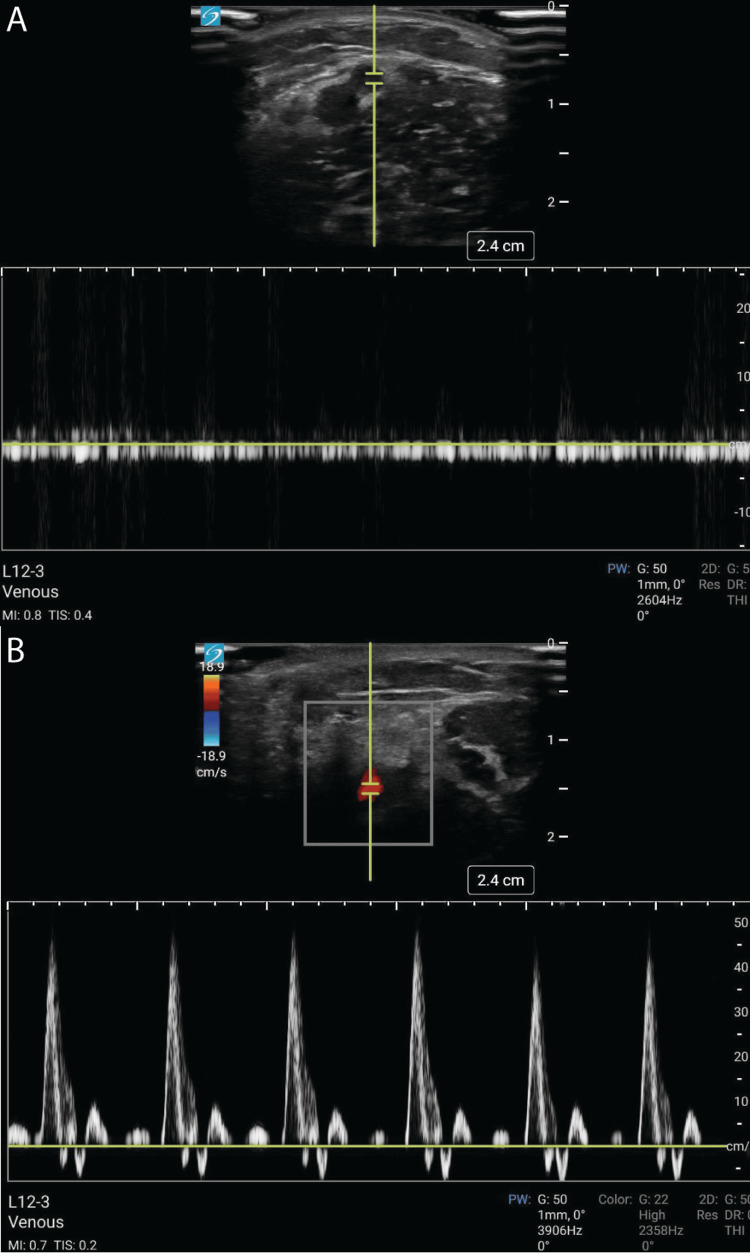
Normal spectral Doppler A) Normal venous spectral Doppler of a radial vein with continuous slow unidirectional flow.  B)  Normal arterial spectral Doppler of the radial artery with multiphasic pulsatile flow.

To evaluate the vasculature, direct pressure is applied to compress the vessels. Normal veins should collapse entirely, and arteries generally collapse with heavy pressure and will demonstrate pulsatility with moderate compression. In some cases, mixed-echogenicity arterial or venous clots can be visualized. Color flow Doppler may also be used to interrogate the vasculature (Video 3); normal veins may demonstrate steady flow and often have increased flow with manual compression of the calf to augment the venous return pressure. Unidirectional pulsatile flow through an artery is easily visualized using color flow Doppler. As in this case, spectral Doppler can also be utilized to more precisely characterize vascular flow. Venous flow on spectral Doppler is slow with little variability in the color signal, while pulsatile arterial flow creates a signal that varies in both color and intensity with each pulse (Figure 4). In cases where flow is not visualized with color flow Doppler or spectral Doppler, power Doppler (which sacrifices directional information in order to maximize sensitivity) can be utilized to evaluate for residual trickle blood flow. These simple techniques allow immediate bedside evaluation for arterial or venous thrombosis and are supported by consensus recommendations [10]; however, POCUS should not be used as a definitive substitute for arterial evaluation in concerning patients. In patients with highly concerning findings, obtaining a more comprehensive imaging study, such as a comprehensive arterial Doppler study or CTA, is recommended.

**Video 3:** Color doppler with normal venous (blue) and pulsatile arterial (red) flow

Expedited diagnosis of arterial thrombosis with POCUS is particularly important due to the time-sensitive nature of acute limb ischemia (ALI). ALI can be categorized with the Rutherford classification scheme [11] described in Table 1. This patient, with impaired sensory and motor function, met the criteria for Category IIb acute limb ischemia. Category IIb patients with severe acute ischemia are at imminent risk of limb loss and permanent disability; emergent revascularization within 12 hours of onset leads to an amputation rate of 6%, while later revascularization between 12 and 24 hours is associated with an amputation rate of 20% [9]. In comparison, Category I ischemia can generally be followed in an outpatient clinic, and Category IIa ischemia can generally be managed with urgent intervention but without emergency surgery. Category III represents irreversible tissue injury, but unless the ischemia is causing systemic illness, there is generally considered to be no benefit to emergent intervention. Given the potential emergent nature of interventions in ALI, rapidly determining the diagnosis of arterial thrombosis with POCUS can be a crucial management step.

**Table 1:** Rutherford limb ischemia classification

Rutherford classification		Exam findings		Doppler signal		
Grade	Category	Sensory loss	Motor deficit	Arterial	Venous	Prognosis
I	Viable	None	None	Audible	Audible	Not immediately threatened
IIA	Marginally threatened	None or minimal (toes)	None	Inaudible	Audible	Salvageable with prompt treatment
IIB	Immediate intervention	More than toes affected	Mild/moderate	Inaudible	Audible	Salvageable with immediate treatment
III	Irreversible injury	Profound, anaesthetic	Profound, paralysis	Inaudible	Inaudible	Inevitable tissue loss or nerve damage

This particular episode of thrombosis was temporally associated with SARS-CoV-2 infection and was especially unusual due to concurrent arterial and venous thrombosis. SARS-CoV-2 infection is associated with a variety of thrombotic events [12], is associated with thrombosis at younger age, without typical cardiovascular risk factors, and has worse outcomes [13-15] but is still not highly associated with concurrent arterial and venous thrombosis [16]. Patients with acute or subacute cases of SARS-CoV-2 should undergo a thorough evaluation for possible thrombotic complications.

## Conclusions

It is essential to maintain a high index of suspicion for arterial thrombosis in patients with SARS-CoV-2 infection. In patients with possible acute limb ischemia, POCUS is the fastest imaging tool available that can expedite evaluation and treatment for this critical diagnosis. Expanding the scope of bedside POCUS evaluation to include arterial evaluation may improve the speed and quality of care for patients in the Emergency Department, but formal protocols and prospective studies are necessary to standardize and optimize care.
